# The drought-tolerant *Solanum pennellii* regulates leaf water loss and induces genes involved in amino acid and ethylene/jasmonate metabolism under dehydration

**DOI:** 10.1038/s41598-018-21187-2

**Published:** 2018-02-12

**Authors:** Isabel Egea, Irene Albaladejo, Victoriano Meco, Belén Morales, Angel Sevilla, Maria C. Bolarin, Francisco B. Flores

**Affiliations:** 1 Department of stress biology and plant pathology, CEBAS-CSIC, P.O. Box 164, 30100 Espinardo-Murcia, Spain; 2 Inbionova Biotech S.L., Edif. CEEIM.University of Murcia, Campus de Espinardo, 30100 Espinardo-Murcia, Spain; 3 Present Address: Department of Molecular Biology and Biochemistry, Instituto de Hortofruticultura Subtropical y Mediterránea, University of Malaga-CSIC, 29071 Malaga, Spain

## Abstract

Breeding for drought-tolerant crops is a pressing issue due to the increasing frequency and duration of droughts caused by climate change. Although important sources of variation for drought tolerance exist in wild relatives, the mechanisms and the key genes controlling tolerance in tomato are little known. The aim of this study is to determine the drought response of the tomato wild relative *Solanum pennellii* (*Sp*) compared with the cultivated tomato *Solanum lycopersicum* (*Sl*). The paper investigates the physiological and molecular responses in leaves of *Sp* and *Sl* plants without stress and moderate drought stress. Significant physiological differences between species were found, with *Sp* leaves showing greater ability to avoid water loss and oxidative damage. Leaf transcriptomic analysis carried out when leaves did not as yet show visual dehydration symptoms revealed important constitutive expression differences between *Sp* and *Sl* species. Genes linked to different physiological and metabolic processes were induced by drought in *Sp*, especially those involved in N assimilation, GOGAT/GS cycle and GABA-shunt. Up-regulation in *Sp* of genes linked to JA/ET biosynthesis and signaling pathways was also observed. In sum, genes involved in the amino acid metabolism together with genes linked to ET/JA seem to be key actors in the drought tolerance of the wild tomato species.

## Introduction

Most crops are susceptible to drought stress, a main environmental factor responsible for yield losses worldwide^1^. The problem of drought stress will worsen in arid and semiarid regions, which are the most affected by the forecasted effects of climate change, and thus pose a serious risk to crop yields and threaten food security^2,3^. In spite of the great efforts invested in crop breeding for drought stress tolerance, the development of tolerant varieties has been slow and is greatly restricted by narrow genetic variations in crops, and by the complex and multigenic nature of the trait of drought tolerance^4,5^. In order to achieve the knowledge required to develop genotypes with enhanced tolerance to drought stress, it is essential to combine the descriptive power of the physiological analysis with the identification of key genes involved in water stress tolerance^6^. Transcriptomic analysis is one useful approach to identify genes playing important roles in drought tolerance and to infer the main mechanisms involved^7,8^. However, it is necessary to take into account that the candidate genes will be different, depending on the drought intensity and the exposure time. Severe water stress during very short periods (hours) will identify genes related to survival as there is no time for the plant to trigger the mechanisms involved in tolerance to long-term. On the other hand, plant response to moderate drought may enable a balance between growth and defence mechanisms under water-limiting conditions^9^, a more real scenario in agronomic conditions, albeit one that is poorly understood^10,11^.

Tomato is considered one of the most economically important horticultural crops grown worldwide^12,13^. What is more, it is an important crop in agriculture in arid and semi-arid zones. Abiotic stresses, like those promoted by water deficiency, have a negative impact on tomato production, particularly in the Mediterranean area^14^. Despite the economic importance of tomato, the mechanisms that govern responses to water stress in this horticultural species are not well characterized, and only a small number of genes playing a role in tomato tolerance to drought have been identified^15,16^. Moreover, despite the wealth of sources of variation for drought tolerance in accessions of tomato wild related species^17^, we still do not know the key genes controlling the tolerance. The wild species *Solanum pennellii* is a drought-adapted species and it constitutes an ideal experimental model to advance in our understanding of the underlying molecular mechanisms of drought adaptation and tolerance in tomato^18^. Moreover, the sequencing of its genome has recently been published and has assisted in the identification of candidate genes involved in stress tolerance^19^. Therefore, *S. pennellii* represents a particularly valuable genetic source for breeding programmes targeted at improving drought tolerance in tomato^18^.

The tolerance of plants to moderate drought stress is the sum of two processes: the plant’s ability to uptake water by the roots, which is generally associated with increased root growth, and its capacity to avoid excessive dehydration of the leaves. However, the beneficial aspects of each process are difficult to separate. The main strategy to date for increasing drought tolerance has been to increase the ability for water uptake of the root system^20,21^. However, the identification of drought-responsive genes expressed above and below ground may enable the elucidation of other potential drought tolerance mechanisms. Previous results from our group showed that one of the strategies used by *S. pennellii* against osmotic stress induced by salinity^22^ was the avoidance of leaf dehydration by osmotic adjustment, inducing high accumulation of solutes during the stress period^23,24^. Interestingly, this adaptation strategy appeared earlier in the time-course of drought response of *S. pennellii* than in cultivated tomato. The tomato wild species accumulated organic solutes as sugars and the diamine putrescine if inorganic solutes were not sufficient to reduce the osmotic potential^24,25^. Another drought tolerant strategy is to limit water loss by leaf transpiration, which is used by *S. pennellii* under osmotic stress, as our previous results also demonstrated^26,27^. Thus, it seems that *S. pennellii* is well adapted against osmotic stress occurring during drought and salinity, showing no visual signs of stress. This tolerance to osmotic stress may be due to either different constitutive gene expression or specific gene expression induction involved in tolerance to this stress.

The comparative study of two phylogenetically closely related tomato species, domesticated *S. lycopersicum* and its wild-relative *S. pennellii*, with a notably different degree of drought tolerance, can increase our knowledge of the genes involved in this tolerance. Furthermore, *S. pennellii* may serve as a model to identify key genes of drought tolerance in the shoot, as the root development of this species is poor and, consequently, its high degree of drought tolerance might be a consequence of molecular processes generated in leaves after stress. In this study we investigated the physiological and molecular responses to moderate drought stress in leaves of cultivated tomato and *S. pennellii*, as the analysis was carried out when the leaves did not show visual dehydration symptoms (after four days of dehydration). Here we show the important constitutive gene expression differences between both species as well as the genes specifically induced by drought stress in the tolerant species *S. pennellii*, which are mainly involved in amino acid metabolism and hormones.

## Results

### The cultivated tomato and the wild species *S. pennellii* show important differences at the physiological level

**Figure 1:**
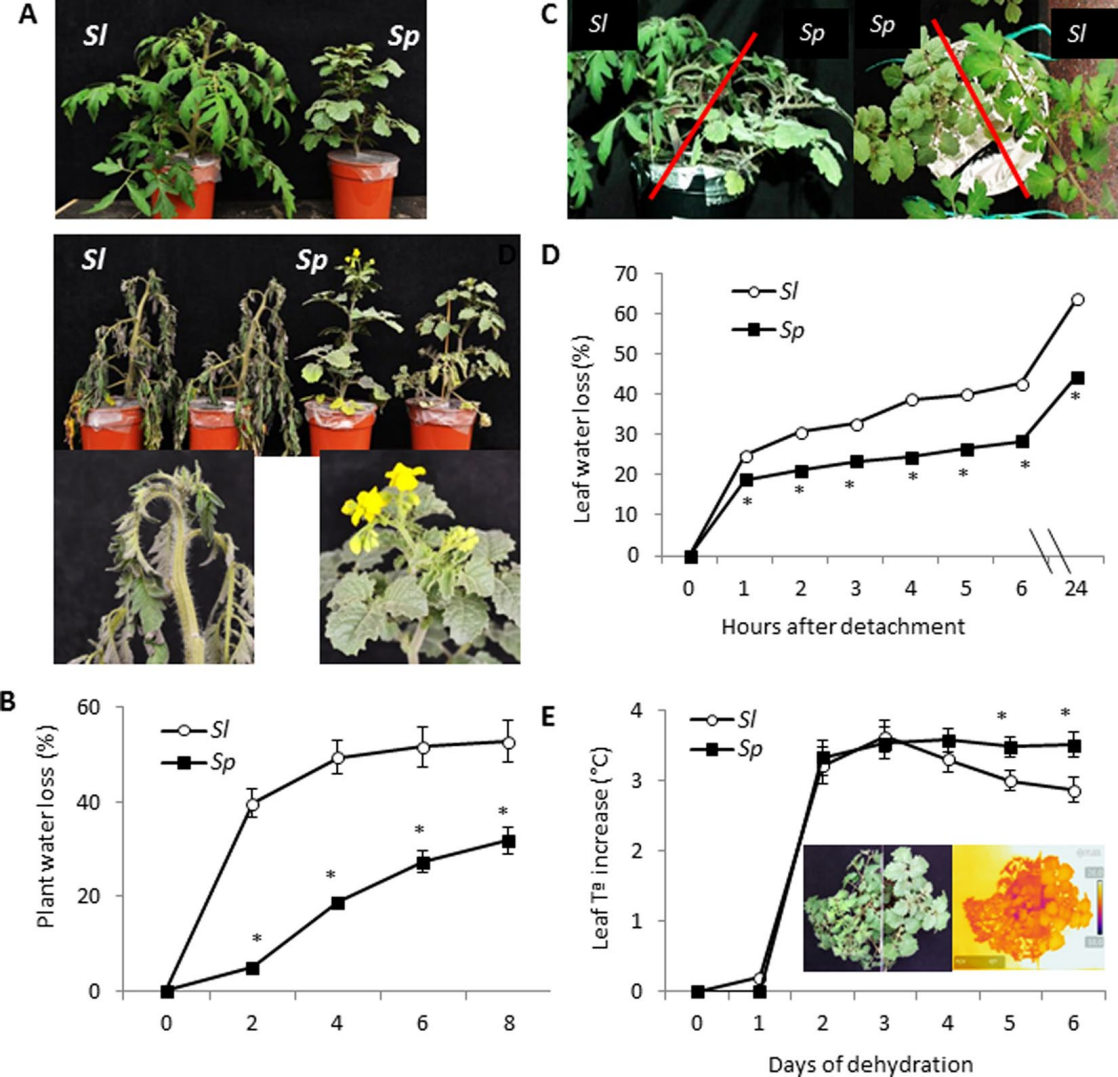
Differences in dehydration degree of cultivated tomato (*Sl*) and the wild species *Solanum pennellii* (*Sp*) subjected to drought stress by withholding water. (**A**) Representative pictures of well-watered plants in individual pots (top) and after 8 days of water withholding (bottom). Pictures of *Sl* and *Sp* apexes at the end of the dehydration cycle are shown below the images of whole plants. (**B**) Plant water loss (transpired water) during the dehydration cycle of plants in separate pots. (**C**) Picture of a pot with *Sl* and *Sp* plants, side-view and from above, after 4 days of withholding water. (**D**) Leaf water loss relative to day 0 from detached leaflets of *Sl* and *Sp* control plants grown in the same pot. (**E**) Changes in leaf temperature (LT) determined by infrared thermography in *Sl* and *Sp* plants placed in the same pot along the dehydration cycle (digital and thermal images at day 4 of water stress treatment shown within the graphic). Results are expressed as mean ± s.e.m., asterisk indicates significant differences between means values of *Sl* and *Sp* (Student t-test) for *P* < 0.05.

When plants of the cultivated tomato (*S. lycopersicum*, *Sl*) and the wild species *S. pennellii*) (*Sp*) were grown in separate pots, visual symptoms after 8 days of dehydration were evident in *Sl* leaves, including the shoot apex, while the leaves of *Sp* showed an aspect more similar to well irrigated plants (control) (Fig. 1A). This difference in visual symptoms was due to the lower transpired water percentages in *Sp* shoots compared with *Sl* throughout the dehydration period (Fig. 1B), which was reflected in the significantly higher shoot water content of *Sp* compared with *Sl* at the end of cycle (3.93 ± 0.06 and 6.41 ± 0.49 mL H_2_O g^−1^ DW in *Sl* and *Sp* respectively). In the following experiment, plants of *Sl* and *Sp* were grown side by side in the same pot, to ensure that both genotypes are exposed to the same severity of water stress^28^ (Fig. 1C). In order to corroborate that the dehydration tolerance of *Sp* is associated to a lower water loss occurring through the leaves, we first measured leaf water loss by using detached leaves from plants grown in control condition (Fig. 1D). Throughout the time of dehydration, leaves of *Sp* plants lost less water than *Sl* from the first hour of detachment, and differences between species held over 24 h. In addition, the leaf temperature (LT) was measured by infrared thermography during the first 6 days of dehydration (Fig. 1E). A significant increase of LT was observed between 1 and 2 days without irrigation in both tomato species. Afterwards, this parameter diverged in both species, decreasing in *Sl* from the 3^rd^ to 6^th^ days while it remained constant in *Sp* during this dehydration period. Representative thermal images of *Sl* and *Sp* plants at the 4^th^ day of dehydration illustrate when *Sp* began to maintain higher LT than *Sl* (Fig. 1E).

**Figure 2:**
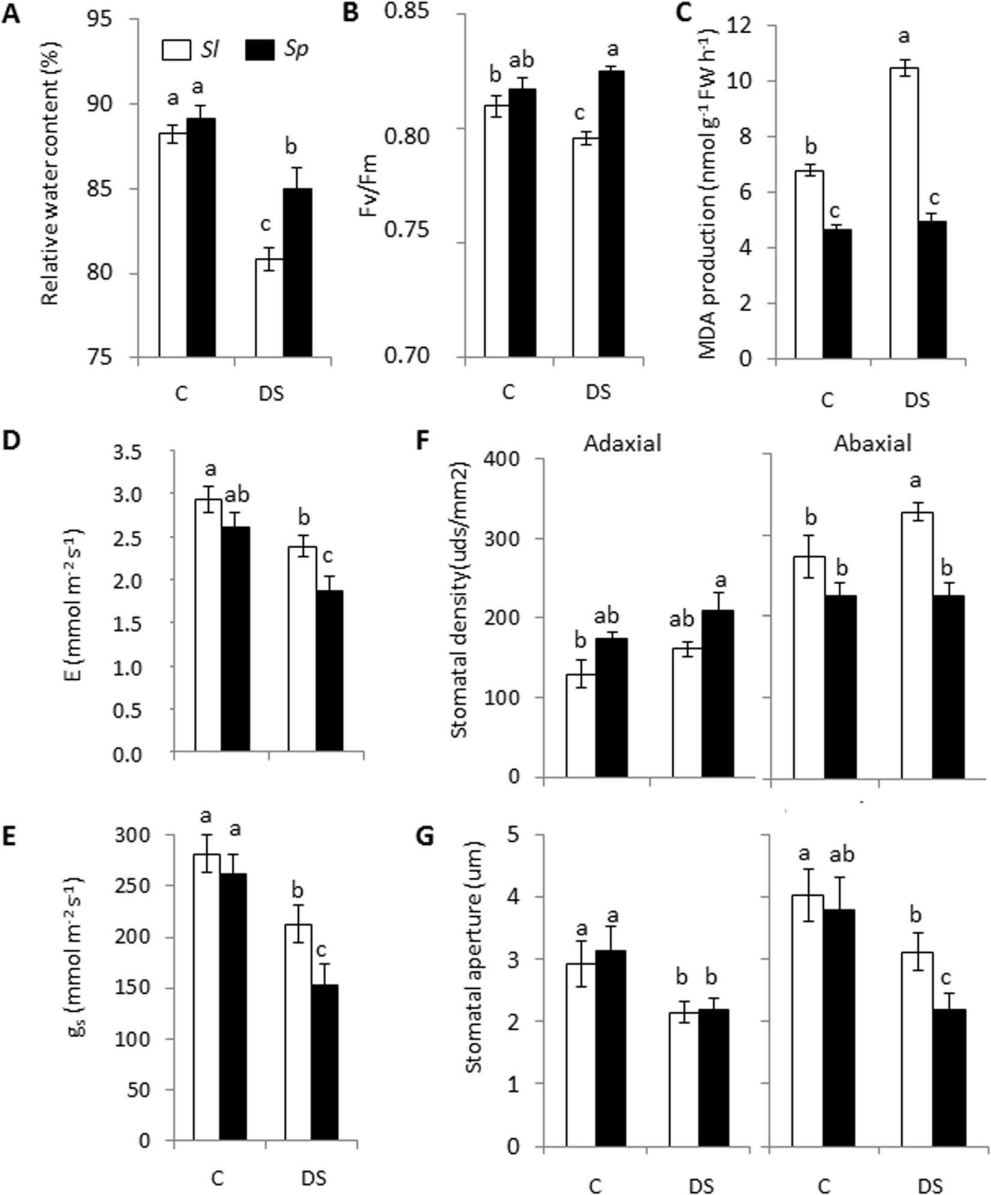
Differences induced by drought stress at the physiological and anatomical levels in leaves of *Sl* and *Sp* plants placed in the same pot. Relative water content (**A**), chlorophyll fluorescence (**B**) and lipid peroxidation degree determined by MDA production (**C**) in leaves of *Sl* and *Sp* plants placed in the same pot, in control (C) and drought stress (DS, 4 days of withholding water). Transpiration rate (E) (**D**) and stomatal conductance (g_s_) (**E**) in C and DS. Stomatal density (**F**) and aperture (**G**) determined in the abaxial and adaxial leaf side of *Sl* and *Sp* plants, in C and DS. Results are expressed as mean ± s.e.m. Values with different letters are significantly different as determined by LSD (*P* < 0.05).

In order to study the differences found between both species at moderate drought stress more deeply, the experiment of dehydration with plants of *Sl* and *Sp* grown in the same pot was repeated again for the first 4 days of dehydration, as at this dehydration time neither the *Sl* nor *Sp* plants showed visual dehydration symptoms yet (Fig. 1C). The values of leaf relative water content (RWC) and leaf PSII photochemical efficiency, measured by *Fv/Fm* ratio, were similar in both species in the control, while they were significantly higher in *Sp* leaves compared with *Sl* under drought stress (Fig. 2A,B). Photosynthetic rate values were similar in both species (12.9 ± 0.8 and 13.6 ± 0.6 µmol m^−2^ s^−1^ in *Sl* and *Sp* at day 0 and 13.2 ± 0.4 and 12.4 ± 0.7 µmol m^−2^ s^−1^ in *Sl* and *Sp* after 4 days of dehydration). In addition, we determined lipid peroxidation measured as malondialdehyde (MDA) production, which is a well-recognized marker for oxidative stress and damage on plants, and we observed that MDA production increased under drought stress only in *Sl* leaves (Fig. 2C). Regarding gas exchange parameters stomatal conductance (g_s_) and transpiration rate (E), significant differences between species were observed only in drought stress, with the values being significantly lower in *Sp* leaves (Fig. 2D,E). In addition, stomatal density and aperture were analysed in the leaf adaxial and abaxial surfaces, since g_s_, a major factor determining the rate of water loss via transpiration, is modulated by both stomatal density and aperture (Fig. 2F,G). Significant changes were found only at the abaxial surface, with both stomatal density and aperture decreasing in *Sp* with respect to *Sl* under drought stress. Taken together, these results hint at the avoidance of water loss through leaves as a main response of *Sp* against drought stress, which is associated to its ability to reduce stomatal density and aperture in the abaxial surface.

### Comparative transcriptomic analysis in leaves of cultivated and wild tomato species

**Figure 3:**
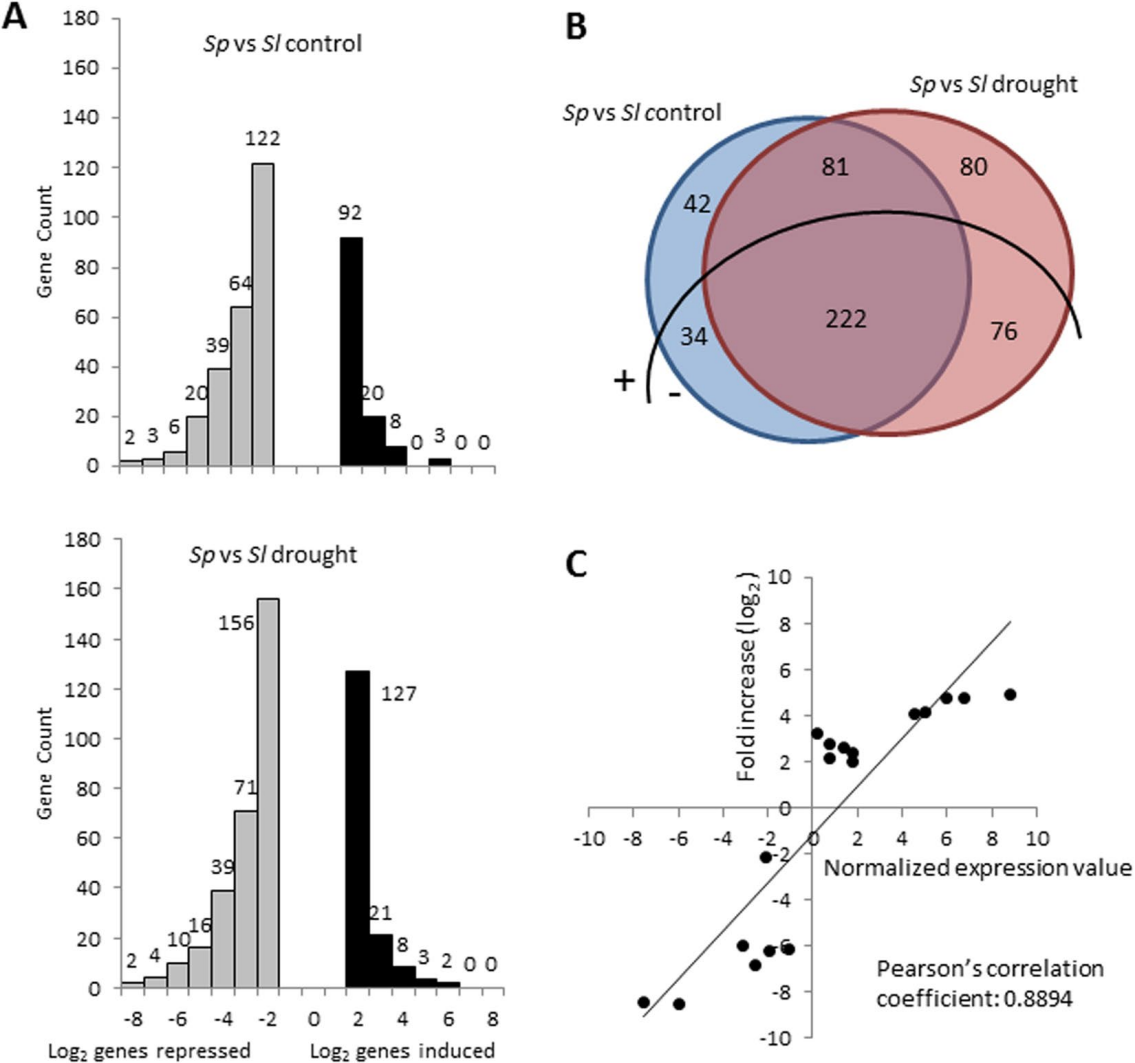
Leaf transcriptome of *Solanum pennellii* (*Sp*) relative to cultivated tomato (*Sl*). (**A**) Distribution of genes induced (black) and repressed (grey) *in Sp* respect to *Sl* in control and drought (after 4 days of dehydration) by the Log_2_ ratio of folds expression. (**B**) Venn diagram showing the number of genes differentially expressed in two comparisons, as well as genes commonly expressed, discriminating up- (+) and down- (−) regulated genes, considering genes showing a FDR < 0.05 and a fold-change threshold value (Log_2_) of 2.0 as differentially expressed genes (DEGs). (**C**) Correlation analysis between microarray (y-axis) and RT-qPCR (x-axis) data. The relative expression values obtained by microarray (Log_2_) were compared with those obtained by RT-qPCR using the ΔΔCt method. The Pearson’s correlation coefficient between relative expression levels is shown. RNA from leaflet tissue of plants grown in control was used as calibrator sample.

The comparative transcriptomic analysis, using the GeneChip® Tomato Genome Array (Affymetrix), was performed on leaves coming from the last water stress experiment. Samples were taken at day 0 (control) and after 4 days of dehydration, when no dehydration symptoms were observed (Fig. 1C). Four comparisons were performed: *Sp* vs *Sl* in control to identify constitutive expression differences between species; *Sp* vs *Sl* in drought stress to identify transcripts affected in *Sp* under stress, which may explain the drought tolerance of *Sp* when compared with *Sl*; *Sp* drought stress vs *Sp* control to identify transcripts affected specifically in *Sp* by drought stress; and *Sl* drought stress vs *Sl* control in order to know whether the *S. pennellii* transcripts are specific. The number of up- and down-regulated differentially expressed genes (DEGs) showing fold changes values between 2.0 and 3.0 outweighed the rest of the DEGs, especially in the case of up-regulated ones, and the number of down-regulated DEGs doubled that of up-regulated ones in both control and drought stress (Fig. 3A). The Venn diagram of the comparison between species showed increases of approximately two-fold in the number of genes expressed only under drought stress compared with those expressed only in control (Fig. 3B). For validation of transcriptomic data, 11 DEGs showing different expression patterns when comparing *Sp* with *Sl* (up- and down-regulated in control and drought stress) were analysed by qRT-PCR (Fig. S1). As shown in Fig. 3C, a significant correlation between the microarray data and qRT-PCR was observed, as the Pearson’s correlation coefficient was 0.8894, which upholds the transcriptomic data.

Each set of DEGs distinguished in the Venn diagram was functionally classified with the MapMan software^29^. In Fig. S2 DEGs from the comparison *Sp vs Sl* are represented, classified in functional categories according to the MapMan software. The functional classifications of up- and down-regulated DEGs in *Sp* compared with *Sl* are shown separately in control, drought stress and both conditions (Tables S1–S6), as well as the up-and down-regulated DEGs in drought stress compared with control, also shown separately in *Sp*, *Sl* and both species (Tables S7–S12). The detailed information about DEGs presented in the following sections is summarized in Table S13.

### Constitutive gene expression differences between cultivated and wild tomato species

**Figure 4:**
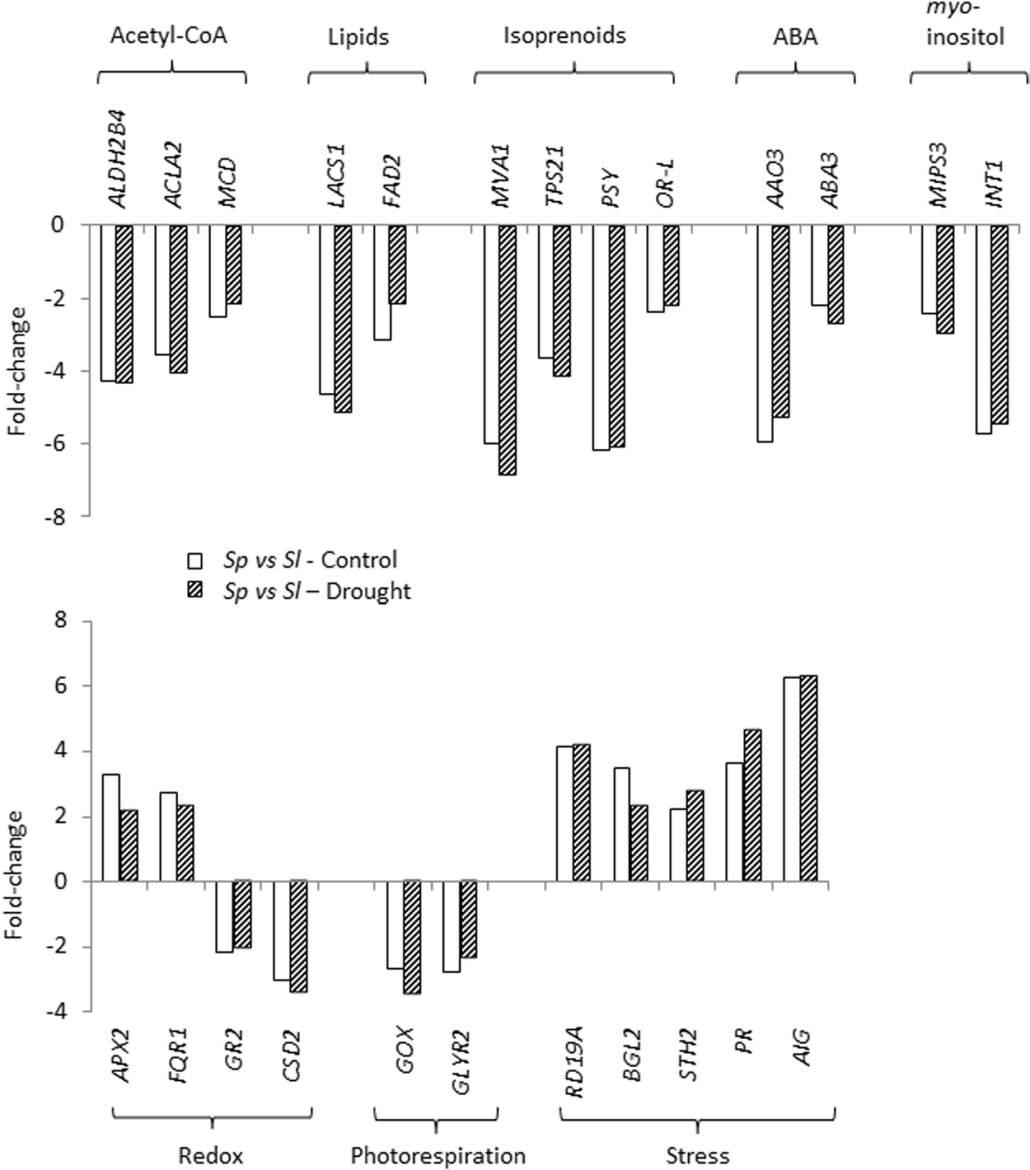
Constitutive expression of differentially expressed genes in *Sp vs Sl*. Genes are linked to different metabolic and physiological processes in leaves of *Sp vs Sl*. Plants were grown in control and drought (4 days of dehydration) conditions. Values of gene expression are represented as fold-changes (Log_2_ scale).

A set of constitutively down-regulated transcripts was found in *Sp* compared with *Sl*, including genes linked to acetyl-CoA production as well as to interconnected pathways of fatty acids and isoprenoid biosynthesis, and all of them were similarly reduced under drought stress (Fig. 4 and Table S5). Among them, it is interesting to point out the gene linked to the carotenoids biosynthesis, upstream of the ABA biosynthetic pathway, *phytoene synthase* (*PSY*). In addition, the *abscisic aldehyde oxidase 3* (*AAO3*) gene involved in the last step of ABA production together with the Arabidopsis ortholog *ABA3*, whose activity is required for AAO functionality, were also down-regulated in *Sp* in both control and drought stress (Fig. 4). Two other DEGs showing similar reduced basal levels in *Sp* with respect to *Sl* in control and drought stress were involved in myo-inositol biosynthesis and transport: *myo-inositol-1-phosphate synthase 3* (*MIPS3*) and myo-inositol transporter *INT1*.

Different changes regarding the expression profile involved in redox homeostasis were detected (Fig. 4 and Tables S2 and S5). The genes encoding for a flavodoxin-like quinone reductase (*FQR1*) and a cytosolic ascorbate peroxidase (*APX2*) were up-regulated in *Sp* compared with *Sl* in both control and drought stress, while genes encoding for the chloroplastic forms of glutathione reductase (GR) and Cu/Zn superoxide dismutase (SOD) showed the opposite response. One source of ROS is the activity of glycolate oxidase (GOX), occurring in peroxisomes during photorespiration^30^. Two genes linked to photorespiration were down-regulated in *Sp* compared with *Sl* in both control and drought stress, *glycolate oxidase* (*GOX*) and *glyoxylate reductase 2* (*GLYR2*) (Fig. 4 and Table S5).

Several stress-responsive genes were up-regulated in *Sp* compared with *Sl*, with quite similar fold-change values in control and drought stress (Fig. 4 and Table S2), as observed in the *RESPONSIVE TO DEHYDRATION 19A* (*RD19A*) gene. When expression analysis using RT-qPCR was performed for *RD19A*, levels were similar to the expression found in the microarray analysis (Fig. S1). There were also DEGs coding for the pathogenesis-related (PR) proteins including *beta 1,3-glucanase 2* (*BGL2*) and *SALT TOLERANCE HOMOLOG 2* (*STH2*), and another coding for a putative PR protein, all of them up-regulated. Another stress-responsive gene highly up-regulated in *Sp* compared with *Sl* in control and drought stress was the Arabidopsis ortholog *AVIRULENCE INDUCED GENE PROTEIN* (*AIG*), which has been described in plant responses to biotic stress.

Finally, a significant number of DEGs with unknown functions were detected in *Sp* in both control and drought stress (Tables S2 and S5). It is important to note the high expression levels found between species of two genes coding for elongation factors (EFs), *GTP-binding elongation factor Tu* (*EF-Tu*) and *Elongation factor 1B gamma* (*EF-1Bg*): the former down-regulated and the latter up-regulated in *Sp* compared with *Sl* (fold-change values of −8.53 and −8.41 for the former in control and drought stress respectively, and 4.97 and 4.82 for the latter). In order to corroborate their high levels of differential expression, both genes were validated by RT-qPCR, confirming the expression patterns from the microarray analysis (Fig. S1).

### Genes induced by drought in the wild species *S. pennellii*

**Figure 5:**
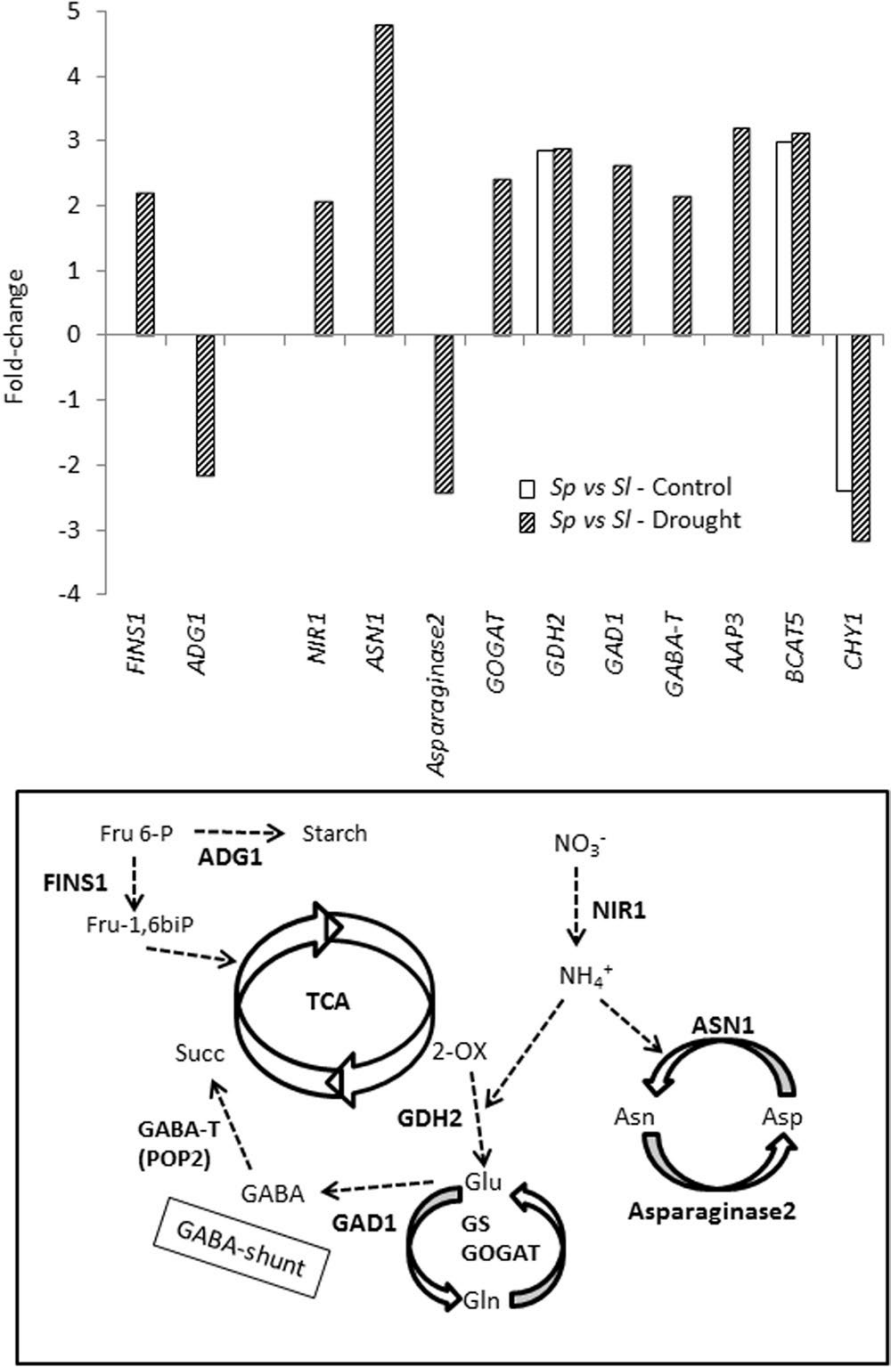
Genes linked to the C/N metabolism are upregulated by drought in leaves of *Sp* compared with *Sl*. Plants were grown in control and drought (4 days of dehydration) conditions. Values of gene expression values are represented as fold-changes (Log_2_ scale).

In relation to the C metabolism, the *FRUCTOSE INSENSITIVE 1* (*FINS1*) gene, whose product is a cytosolic fructose-1,6-bisphosphatase, was specifically up-regulated in *Sp* compared with *Sl* under drought stress. In contrast, the gene involved in starch biosynthesis *ADP-glucose pyrophosphorylase 1* (*ADG1*) was down-regulated (Fig. 5 and Tables S3 and S6), which suggests that *Sp* prevents a shift of carbon allocation towards starch synthesis.

A remarkable number of genes involved in N metabolism were up-regulated in *Sp* respect to *Sl*, and most of them were only induced under drought stress (Fig. 5, and Tables S2, S3, S5 and S6). Thus, two genes involved in N assimilation, *nitrite reductase 1* (*NIR1*) and *glutamine-dependent asparagine synthase 1 (ASN1)*, were induced by drought stress in *Sp*. When expression analyses using RT-qPCR were performed for these two genes, *NIR1* and *ASN1*, results confirmed the expression patterns of the same genes from the microarray analysis (Fig. S1). Importantly, one way of reducing the excess NH_4_^+^ caused by stress is to integrate it in the amino acid metabolism, and besides *ASN1* induction, the gene *asparaginase 2* involved in the reverse reaction (the production of aspartate from asparagine and release of NH_4_^+^) was specifically down-regulated by drought stress in *Sp*. Another gene up-regulated in *Sp* compared with *Sl* is *NADH-dependent glutamate synthase 1*(*GLT1*), whose gene product is a chloroplastic NADH-dependent glutamate synthase (also known as glutamine-oxoglutarate aminotransferase, GOGAT). Another metabolic way of assimilating most inorganic N, especially excess NH_4_^+^ occurring in stressful conditions, is through the glutamine synthetase/glutamate synthase (GS/GOGAT) cycle. A DEG coding for the Arabidopsis ortholog *glutamate dehydrogenase 2* (*GDH2*) was already induced at basal level in *Sp* respect to *Sl* and was maintained in drought stress. In addition, genes involved in the ϒ-aminobutyric acid (GABA) metabolism, *glutamate decarboxylase 1* (*GAD1*) and *ϒ-aminobutyrate transaminase* (*GABA-T*), were specifically induced by drought stress in *Sp* compared with *Sl*. A DEG coding for an amino acid transporter, the Arabidopsis ortholog *amino acid permease 3 AAP3*, has been found to be stress-responsive and it is markedly up-regulated in *Sp* compared with *Sl*, which may intervene in the amino acids transport among cytosol, mitochondrion and chloroplast, where the above mentioned gene products exert their functional activity. Moreover, a gene involved in branched-chain-amino-acid biosynthesis, the *branched-chain-amino-acid transaminase 5* (*BCAT5*), was induced in *Sp* both in control and drought stress, while the opposite response was found in the *3-hydroxyisobutyryl-CoA hydrolase 1* (*CHY1*) gene involved in BCAA catabolism. Taken together, the strategy used by the wild species *Sp* to respond to drought stress is to induce the gene expression linked to N metabolism, as it is schematically shown (Fig. 5).

**Figure 6:**
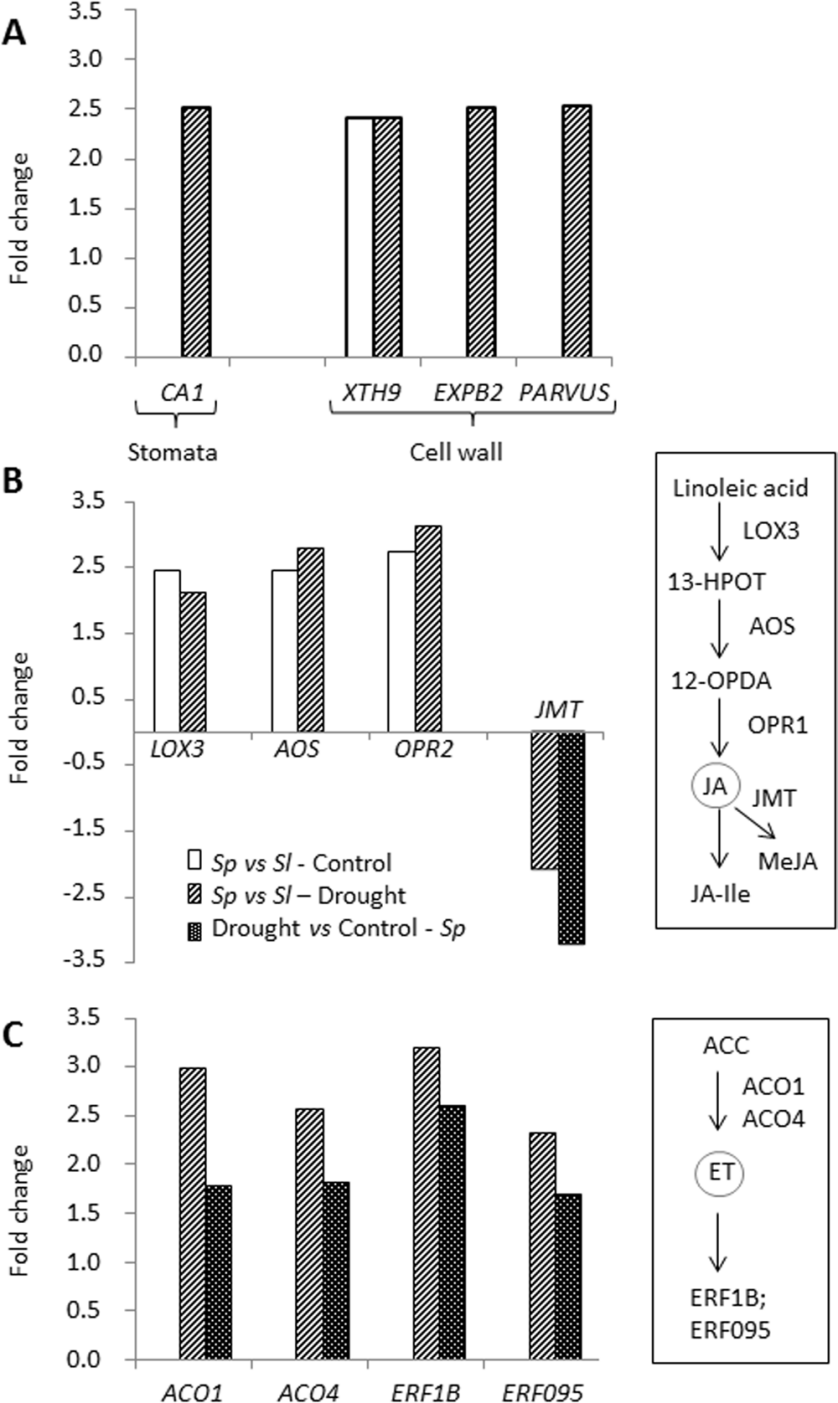
Induction of differentially expressed genes in *Sp vs Sl* and by drought in *Sp*. Genes linked to stomata movement and cell wall physiology (**A**), and JA (**B**) and ET (**C**) metabolism are upregulated by drought in leaves of *Sp* with respect to *Sl*. Plants were grown in control and drought (4 days of dehydration) conditions. Values of gene expression are represented as fold-changes (Log_2_ scale).

In relation to leaf water loss, the *carbonic anhydrase 1* (*CA1*) gene was induced by drought stress in *Sp* (Fig. 6A and Table S3), which is in accordance with the water loss reduction due to reduced stomatal density and aperture (Fig. 2F,G). In addition, drought stress-responsive genes involved in cell wall metabolism were also up-regulated by drought stress in *Sp* (Fig. 6A and Tables S2 and S3), as *XTH16* (*xyloglucan endotransglucosyl/hydrolase 16*), already induced at the basal level and *EXPB2* (*expansin B2*), both linked to cell wall extensibility. Moreover, the *PARVUS/GLZ1* gene involved in cell wall thickening was up-regulated by drought stress in *Sp*.

Regarding hormones metabolism, different genes involved in JA and ET were up-regulated in *Sp* compared with *Sl*. Thus, genes involved in JA biosynthesis *lipoxygenase 3* (*LOX3*), *allene oxide synthase* (*AOS*) and *OPDA reductase 2* (*OPR2*) were up-regulated in *Sp* compared with *Sl* in both control and drought stress (Fig. 6B and Table S2). However, *jasmonic acid carboxyl methyltransferase* (*JMT*) gene, whose product catalyses the conjugation of JA to form the non-bioactive compound methyl jasmonate (MeJA), was down-regulated by drought stress in *Sp vs Sl*, as well as in the comparison between drought stress *vs* control of *Sp* (Fig. 6B and Tables S6 and S10). Regarding ET biosynthesis and signaling, it is notable that genes induced by drought stress in *Sp vs Sl* were also up-regulated when comparing drought stress *vs* control in *Sp* (Fig. 6C and Tables S3 and S7). Thus, two *ACC oxidases* (*ACO1* and *ACO4*), involved in the last step of ET biosynthesis, and the *ethylene-responsive element binding factors ERF1B* and *ERF95* were all up-regulated in both comparisons, which suggests that ET biosynthesis and signaling are important issues in the drought response of the wild tomato species.

Finally, as in the case of genes with constitutive expression, we found a notable proportion of up-regulated DEGs with unknown functions, achieving percentages of around 20% with respect to the total DEGs in both comparisons, *Sp vs Sl* in drought stress, and drought stress compared with control in *Sp*.

## Discussion

Differences in tolerance mechanisms between wild and cultivated tomato species may result from changes in constitutive basal levels of genes involved in different processes^17^. In the wild species *Sp*, genes involved in acetyl-CoA and the branched pathways of fatty acids and isoprenoids biosynthesis, as well as genes linked to *myo*-inositol and photorespiration, were constitutively down-regulated, compared with *Sl*, and these differences were maintained under drought (Fig. 4). The lower expression of genes in *Sp* could be due simply to a higher requirement of these essential components of the metabolism in domesticated tomato, since the trend of gene expressions is generally the same in control and drought. However, it could also point to a more efficient metabolism in a species where its natural habitat is already stressed. Thus, in photorespiration there is a particular reaction responsible for the highest amount of H_2_O_2_ produced in this metabolic pathway, which must be tightly regulated for the sake of redox homeostasis, which is the oxidation of glycolate to glyoxylate in peroxisome generating H_2_O_2_, making this organelle one of the main intracellular sites of ROS production^30^. The fact that the expression of *GOX*, involved in the conversion of glycolate into glyoxylate, was constitutively down-regulated in *Sp* compared with *Sl* suggests that *Sp* is able to reduce the H_2_O_2_ and glyoxylate accumulation. Moreover, the presumably lower amount of GOX present in *Sp* evidences the lack of need to activate the alternative route of glyoxylate conversion catalysed by GLYR, as the constitutive down-regulation of *GLYR2* suggests (Fig. 4).

### The wild species *S. pennellii* shows high ability to prevent leaf water loss

The changes observed at the physiological level demonstrate the ability of *Sp* to avoid leaf water loss under drought conditions, as *Sp* reduced g_s_ and E compared with *Sl*, and *Sp* plants exhibited lower stomatal density and aperture in the leaf abaxial surface (Fig. 2D–G). Taking into account that the regulation of stomatal degree of aperture and density in the leaves must be highly controlled for the plant to be able to adapt to stressful conditions^31^, this response of *Sp* seems to be generalized to the osmotic effects induced not only by drought but also by other abiotic stress. Thus, we recently observed that *Sp* increased the water content of their leaves by reducing stomatal density and aperture under salinity^27^.

Numerous studies point out the importance of cell wall in limiting the water loss under drought^32,33^. In this sense, the ability of *Sp* to prevent leaf water loss might be associated with the up-regulation of genes involved in cell wall extensibility, like *XTH16* and *EXPB2*, and cell wall thickening, like *PARVUS/GLZ1* (Fig. 6A). In relation to water loss through stomata, carbonic anhydrases (CAs) are main regulators of stomatal movement and density, inducing stomatal closure^34–36^. In photosynthetic tissues, CA contributes to mesophyll conductance (g_m_) by maintaining the CO_2_-HCO_3_^−^ equilibrium in the cytosol and chloroplasts, so facilitating access to CO_2_ for fixation by Rubisco^37,38^. Recently, Momayyezi and Guy^39^ indicated that CA activity plays a significant role in mediating the mesophyll resistance to CO_2_ diffusion in black cottonwood. Our study hints that the wild species is trying to reduce water loss by inducing the closure of stomata via CA, as the drought-specific upregulation of *CA1* gene suggests, which is in line with the reduction of stomatal density and aperture as well as g_s_ and E found in this wild tomato species in water stress (Fig. 2D–G).

The strategy of modifying cell wall properties or closing stomata in order to avoid water loss must be balanced since it triggers a concomitant reduction of CO_2_ uptake, affecting plant growth^40,41^. Recently, Onoda *et al*.^42^ indicated that a greater fraction of leaf mass in cell walls is associated with a lower fraction of leaf N invested in photosynthetic proteins, and lower within-leaf CO_2_ diffusion rates due to thicker mesophyll cell walls. In addition, stomatal closure may disturb the redox homeostasis^30^. However, *Sp* seems to be able to achieve a trade-off between water loss and growth maintenance. Thus, *Sp* suffers a lower degree of oxidative stress according to the MDA levels, an indicator of oxidative stress, as the values increased with drought stress in *Sl* but not in *Sp* leaves (Fig. 2C). Furthermore, the *APX2* gene was induced in *Sp* with respect to *Sl*, which favours the maintenance of redox homeostasis in the wild tomato (Fig. 4). On the other hand, C depletion as consequence of stomatal closure does not seem to occur in *Sp* but rather the opposite, according to the upregulation of *FINS1* gene, involved in the synthesis of Fru-6-P, together with the downregulation of *ADG1* gene, involved in the starch biosynthesis (Fig. 5), which suggests that *Sp* is preventing a shift of C allocation towards starch synthesis in order to mobilize sugars. Furthermore, the down-regulation in *Sp* of *MIPS3* gene (Fig. 4), which codifies for the rate-limiting enzyme in the synthesis of myo-inositol^43^, seems to contribute to the avoidance of deviating C towards others metabolites, as myo-inositol is derived from glucose-6-phosphate.

### Genes involved in amino acid metabolism and JA/ET are induced by drought in the wild species *S. pennellii*

Although maintenance of C/N balance in plant metabolism is critical in plant growth and has to be finely regulated in stressful conditions^44^, to date little is known about the regulation of genes involved in N metabolism in *Sp* under drought. Interestingly, an important number of genes involved in N metabolism were up-regulated by drought in *Sp* (Fig. 5), as *GDH2* and *ASN1*, involved in the synthesis of Glu and Asn, respectively. The production of Asn is a main way for N transport and storing in higher plants, since this amino acid has a high N/C ratio and is very stable^45^, with ASN playing a key role in the response to diverse stresses^44,46^. It is interesting to point out that the high expression level of *ASN1* in drought-stressed leaves of *Sp* was accompanied by inhibition of *Asparaginase2*, whose product catalyses the opposite process. In addition, the *Sp* tolerance also seems to be related to induction of the GOGAT/GS cycle and the GABA-shunt, as *GLT1,GAD1* and *GABA-T* are specifically induced by drought stress in *Sp*. The activation of the GABA-shunt stimulates the conversion of Glu into succinate ready to be used by the TCA cycle, so contributing to maintain the C/N balance in *Sp* under drought conditions. Michaeli *et al*.^47^ confirmed the importance of GABA as a precursor for multiple metabolic pathways, including the TCA cycle and polyamine metabolism. In this respect, previous studies showed that the main differences between the two tomato species lie in an earlier and greater accumulation of putrescine in *Sp* than in *Sl* under salt stress, and these changes were associated with changes in the amino acids levels related to its synthesis as Glu^24^. Although there are a number of studies demonstrating involvement of the GABA shunt in response to abiotic stresses^48^, information on the molecular mechanism underpinning the role of this pathway in the drought tolerance of *Sp* is practically non-existent. It is interesting to note that in a recent study carried out with tomato, the drought tolerance induced by paclobutrazol application was related to increased expression of genes involved in TCA cycle and GABA shunt^49^. In sum, a higher replenishment of the TCA cycle through activation of the GS/GOGAT cycle and the GABA-shunt seems to be associated to the drought tolerance of *Sp*. Finally, it is interesting to point out that increased N assimilation may improve water status of the plant via reductions in g_s_ and E^50,51^, such as has been observed in *Sp* under drought (Fig. 2D,E).

Hormones are central integrators that link and control the complex stress-adaptive signaling cascades^52^. JA is considered a key regulator of expression of stress-responsive genes in virtually any plant species^53–55^. Recently, we identified the *res* (*restored cell structure by salinity*) tomato mutant that has constitutively activated the JA biosynthesis pathway, and it exhibits tolerance to abiotic stresses^56,57^. Interestingly, we observed that genes from the JA biosynthesis pathway, such as *LOX3*, *AOS* and *OPR2*, are constitutively up-regulated in *Sp* compared with *Sl*, and differences were maintained in drought stress (Fig. 6).

Another hormone involved in the plant response to abiotic stress is ET^58^. Note that genes involved in ET biosynthesis and signaling, specifically in the steps catalysed by ACO and regulated by ERFs, are induced by drought stress in *Sp* leaves (Fig. 6). Interestingly, Phukan *et al*.^59^ indicated that ERFs regulate the expression of a wide variety of down-stream target genes related to stress response and development through different mechanisms, and in our study both *ERF1B* and *ERF095* are up-regulated in *Sp*. Although the role of ERFs in hormone cross-talk under drought is still scarcely known^60^, it has been suggested that *ERF1* induction requires ET as well as JA signaling under different abiotic stress conditions^61,62^. In addition, it has been shown that ET/JA signaling is also required for the induction of other *ERFs* in response to abiotic stresses^63^, as observed in *ERF095* (Fig. 6). Regarding the oxidative stress, overexpression of *LeERF1* and *LeERF2* reduced MDA levels in tomato plants under salt stress^64^. Our results are in concordance with those obtained in tomato, as the MDA values were lower in *Sp* leaves compared with *Sl* ones in drought stress (Fig. 2C). On the other hand, *ERF1* seems to activate specific sets of stress-responsive genes by targeting their DRE elements during abiotic stress, among them the responsive to dehydration *RD20* and *RD29B* genes^62^. In our study the expression of *RD19A* was induced in *Sp* compared with *Sl*, although this is constitutive (Fig. 4). Taken together, the induced expression of ET metabolism/signaling genes in *Sp* seems to be related to its drought tolerance.

**Figure 7:**
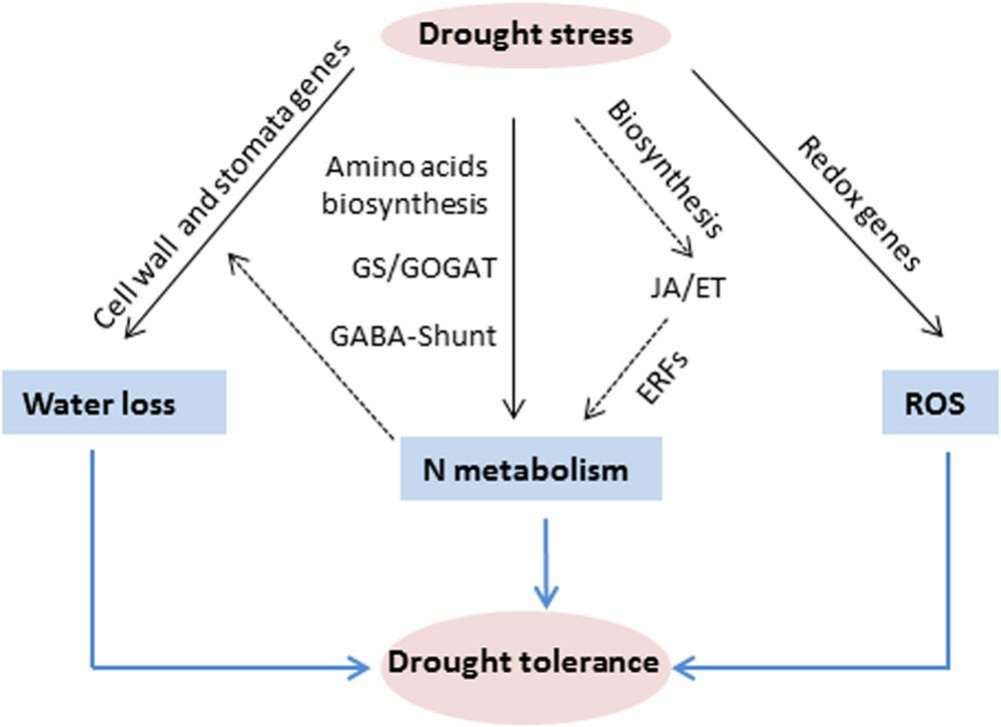
A proposed model for genes playing important roles in processes that regulate drought tolerance of *Sp*. Under drought, *Sp* reduces water loss by upregulation of genes involved in cell wall in conjunction with the stomatal regulator *CA1* gene. At the same time, the low degree of oxidative stress that suffers *Sp* under drought stress is associated to the induction of genes involved in redox homeostasis, as *APX2*. In addition, genes linked to different processes of N metabolism and JA/ET pathways are induced by drought in *Sp*, in the manner that the downstream genes (*ERFs*) could participate in N metabolism, which in turn might improve the leaf water loss.

It has been pointed out that ET/JA among other hormones signaling components regulates the trade-off between plant growth and stress tolerance^65–67^. One facet of this regulation occurs when interacting with the N metabolism, as observed by Zhang *et al*.^68^ in roots. Our results for *Sp* leaves also hint in this direction, as genes involved in amino acid metabolism together with genes linked to ET/JA biosynthesis-signaling are the main ones induced by drought in the drought-tolerant species (Figs 5 and 6). Based on the overall results, a model of the different processes and genes involved in the *Sp* drought tolerance response was proposed (Fig. 7). Besides the coordinated regulation of genes involved in leaf water loss, redox homeostasis and N metabolism, genes linked to ET/JA pathways and the downstream ERFs may participate in N metabolism, which in turn may improve the leaf water loss. Although the existence of other mechanisms involved in regulating drought tolerance in *Sp* is not excluded, it is evident that the different genes identified in *Sp* leaves may be potentially important players in the drought response of the wild tomato.

## Material and Methods

### Plant material and drought stress assays

Cultivated tomato (*Solanum lycopersicum* L.) cv. P73 and the wild species *Solanum pennellii* (Corell) accession PE47 (collected in Peru by Cuartero *et al*.^69^ were used in all experiments. Seeds were germinated in darkness in a 2:1 (v/v) mixture of peat:perlite, at 28 °C temperature and 90% relative humidity. The germination of *S. pennellii* was started 8 days earlier than cultivated tomato in order to get plants in a similar developmental stage when ready for the water stress experiments. Plant culture was carried out in a controlled conditions growth chamber, and the environmental conditions were 18–25 °C, 50–80% of relative humidity, and a photoperiod 16 h light/8 h dark. A photosynthetic photon flux density (PPFD) of 345 μmol m^−2^ s^−1^ at the plant level was provided by fluorescent tubes (Osram Lumilux daily-light 58 W and Fluora 58 W). Seedlings were transferred to plastic pots filled with peat and perlite (2:1), and were daily irrigated with half-strength Hoagland solution^70^.

Two different procedures were used for drought stress assays. In the first experiment plants of each species were placed in separated pots of 1.5-L capacity, while the two other experiments were carried out by placing one plant of each species in the same pot, although the volume of the pots was double (3-L). In all experiments, the control plants were irrigated daily up to pot capacity, and drought stress was applied by withholding irrigation, so water stress intensity increased with time of dehydration cycle. The drought stress was applied when the 4^th^ true leaf was expanded. In order to avoid water evaporation from substrate, the pots were covered with parafilm, leaving room for only the plant main stem to protrude.

Three replicates per species and treatment were used in all experiments. Samples from the first two true leaves were harvested between 4–5 h after the beginning of the light period for physiological and molecular analyses. A pool was prepared from leaflets of these two leaves per sample and half of them were used for physiological analysis, and the other half were immediately frozen in liquid nitrogen and stored at −80 °C.

### Physiological measurements

Plant water loss (transpired water) during dehydration cycle was determined as a percentage of the difference between the pot weight for each time and for day 0. Leaf water loss was determined in detached leaflets as described in Campos *et al*.^71^. Infrared thermography imaging and leaf surface temperatures measurements were performed following procedures described in Albaladejo *et al*.^27^. Gas exchange parameters, stomatal conductance (g_s_) and transpiration rate (E) were determined with an Infrared Gas Analyser (IRGA), following Campos *et al*.^71^. To assess the photochemical efficiency of photosystem II (PSII), chlorophyll fluorescent (*Fv/Fm*) was determined with a portable Chlorophyll Fluorometer (Opti-Sciences, Hudson, NH), following the protocol described in Garcia-Abellán *et al*.^56^. Stomatal density and aperture were measured by light microscopy, by means of the protocol described in Albaladejo *et al*.^27^.

Shoot water content was determined for the whole shoot (leaves and stems) according to the formula (FW − DW)/DW, where FW is the fresh weight and DW the dry weight obtained oven dried for 48 h at 80 °C. The leaf relative water content (RWC) was determined as (FW − DW)/(SW − DW), where SW is the saturation weight determined after 24 h re-saturation in tap water. Lipid peroxidation was determined to assess the impact of secondary oxidative stress in plants subjected to water stress. The thiobarbituric acid reactive substrates (TBARS) assay was used as previously described^72^.

Data were statistically analysed using ANOVA, with means separated by Student’s *t*-test (*P* < 0.05). The effects of treatment and tomato species were analysed using two-way ANOVA, with means separated by least significant difference (LSD) (*P* < 0.05). All data are given as mean ± standard error (s.e.m.). Significant differences between means are denoted by different lower case letters or asterisks.

### Microarray hybridization and data analysis

Microarray hybridization was performed by inBIOnova Biotech S.L., a start-up company sited at the campus of the University of Murcia. RNA isolated from leaflets coming from three individual pooled plants (biological replicates) was used in hybridization to one chip, resulting in total 12 chips (three replicates per each species and treatment). RNA extraction was performed with RNeasy Mini kit and QIAshredder (Qiagen) following the manufacturer’s instructions, and the amount and quality of the RNA checked by Bioanalyzer and spectrophotometrically by Nanodrop. Biotinylated cRNA was synthetized from 200 ng of each sample using the GeneChip 3′ IVT Express kit (Affymetrix), according to the protocol supplied by the manufacturer. The amount and quality of biotinylated cRNA was checked by Nanodrop and agarose gel electrophoresis. The biotinylated cRNA targets were cleaned up and 15 µg were fragmented in order to prepare the hybridization mix, using the Hybridization, Wash and Stain kit (Affymetrix) according to recommendations of manufacturer. The resulting preparations were hybridized to GeneChip® Tomato Genome Array (Affymetrix) interrogating 10209 sequences. After applying hybridization (spike controls) and labelling (polyA controls and 3′/5′ ratios of housekeeping genes) tests, it was observed that the 12 chips have fulfilled the quality criteria.

With the aim of minimizing the discrepancies due to variables such as sample preparation, hybridization, etc. all the arrays were scaled by defining an arbitrary value of 500 as average intensity and using the Expression Console^TM^ (EC 1.1, Affymetrix®) software. The differences of scaling factor among the different samples did not exceed 3 in any case, so we conclude the results are sufficiently homogenous and feasible as required. The BioConductor affyPLM package was used to obtain Normalized Unsealed Standard Error (NUSE) plot to study the quality of the chips for each set of probes from intensity values of each probeset. All samples presented NUSE values within the required ranges. The intensity value of each probe in the array was processed and normalized according to the Robust Multichip Average (RMA) method to obtain an individual intensity value for each probeset. First filtering, normalization and second filtering of probes yielded a final list of 5050 sequences (working list). Non-supervised Principal Components Analysis (PCA) and hierarchical clustering were performed and showed that samples tend to separate according to genotype and condition. Differentially expressed genes (DEGs) were identified by the LIMMA test^73^, which was corrected for multiple test using the FDR^74^ on the working list. Genes with a FDR < 0.05 and a fold change (Log_2_ ratio value) of ≥2.0 when comparing genotypes in the same experimental condition were identified as DEGs. A final list of 535 genes was obtained with the above conditions. The annotation of probe sets was obtained from Affymetrix and loaded in the Partek Genomics Suite software (Partek Incorporated, St. Louis, USA). For the functional study of DEGs MapMan software was used^29^ since it has a more completed database for functional assignations. The Slyc_AFFY_SGN_BUILD2_070709 database was loaded and used for this functional analysis. The statistical analysis followed was of Willcoxon Rank Sum test with Benjamini-Hochberg correction.

### Data deposition

The microarray data and the related analysis information from this research work were deposited in the Gene Expression Omnibus (http://www.ncbi.nlm.nih.gov/geo/) under accession number GSE97045.

### Quantitative real-time PCR verification

The expression profiles obtained from microarray hybridization were further validated by RT-qPCR, using a set of 11 DEGs randomly selected (Table S14). For gene expression analysis, total RNA extracts isolated from leaflets coming from three individual pooled plants (biological replicates) from the microarray experiment were used. Total RNA was quantified in a GeneQuant II spectrophotometer (Pharmacia Biotech) and 5 μg were used for cDNA synthesis with First Strand cDNA Synthesis Kit (Thermo Fisher Scientific). Quantitative real-time RT-qPCR was carried out as described by Garcia-Abellan *et al*.^75^ using 1 μL of undiluted cDNA mixed with iTaq™ Universal SYBR® Green Supermix (Bio-Rad) and 0.45 μL of forward and reverse primers in a CFX Connect^TM^ Real-Time PCR System (Bio-Rad) cycler. Serial dilutions of cDNA were used to make a standard curve to optimize amplification efficiency (Table S14). No template controls were included. All reactions were performed in triplicate with three different RNA extracts for each sample. The presence of a single band on an agarose gel electrophoresis and of a single peak in the melting temperature curve confirmed the specificity of qRT-PCR amplification. Relative expression data were calculated as described by Asins *et al*.^76^ using the tomato elongation factor 1α (*LeEF1α*, acc. AB061263) as housekeeping gene. Gene expression was quantified by the comparative method (2^−ΔΔCt^)^77^, using the expression level of each gene from leaflet tissue of plants grown in control condition as the calibrator sample.

## Electronic supplementary material

Supplementary Figures S1 and S2, and Supplementary Tables S13 and S14

Supplementary Tables: Functional Classification of Differentially Expressed Genes
