# Supplementary material for: The drought-tolerant Solanum pennellii regulates leaf water loss and induces genes involved in amino acid and ethylene/jasmonate metabolism under dehydration
Source: Sci Rep. 2018 Feb 12;8:2791. doi: 10.1038/s41598-018-21187-2 (PMC5809557; doi:10.1038/s41598-018-21187-2)
Supplement: Supplementary file 1 — Supplementary Figures S1 and S2, and Supplementary Tables S13 and S14 [file 41598_2018_21187_MOESM1_ESM.pdf]

The drought-tolerant *Solanum pennellii* regulates leaf water loss and induces genes involved in amino acid and ethylene/jasmonate metabolism under dehydration

Isabel Egea[1+], Irene Albaladejo[1+], Victoriano Meco[1#], Belén Morales[1], Angel Sevilla[2], Maria C. Bolarin[1] & Francisco B. Flores[1]*

[1]Department of stress biology and plant pathology Dpt., CEBAS-CSIC, P.O. Box 164, 30100 Espinardo-Murcia, Spain.
[2]Inbionova Biotech S.L., Edif. CEEIM. University of Murcia, Campus de Espinardo. 30100 Espinardo-Murcia, Spain
[#]Present address: Department of Molecular Biology and Biochemistry, Instituto de Hortofruticultura Subtropical y Mediterránea, University of Malaga-CSIC, 29071 Malaga, Spain.
[+]These authors contributed equally to the work

*Corresponding author:
borjaflores@cebas.csic.es

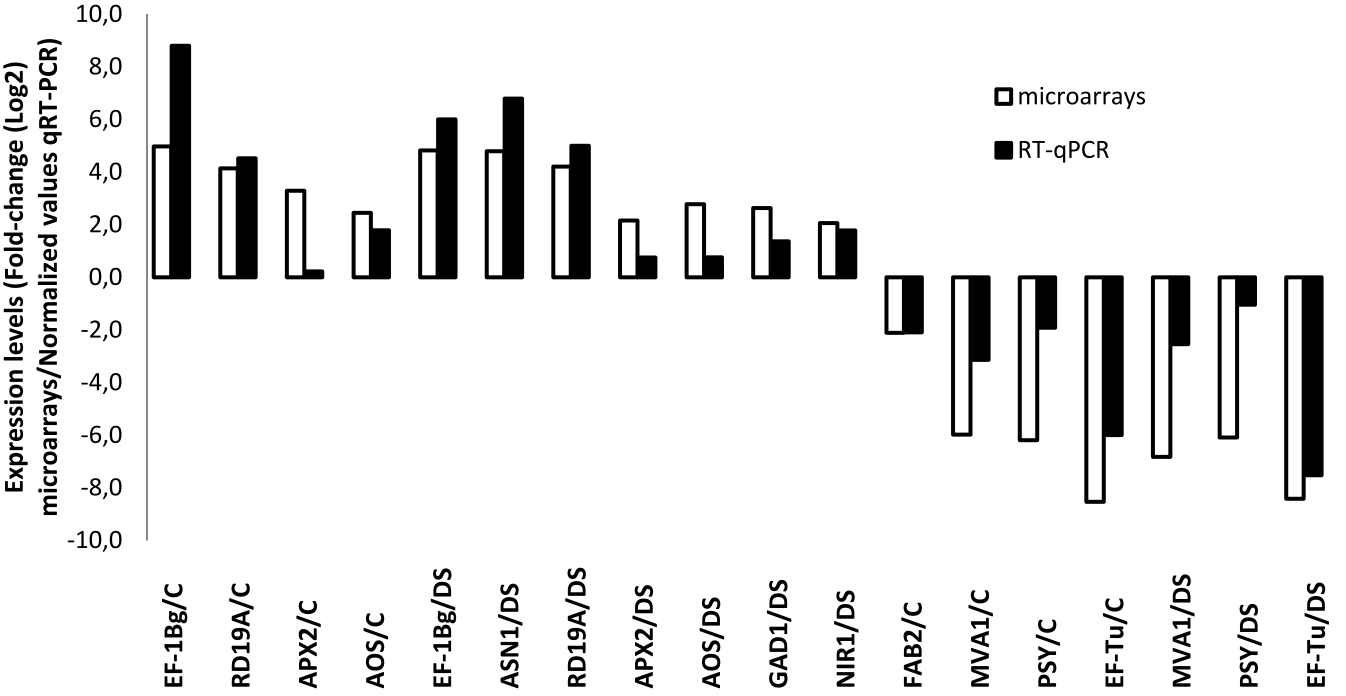

Supplementary Information Figure S1. RT-qPCR validation of microarray data on 11 selected genes (Supporting Information Table S13) from *S. pennellii* and *S. lycopersicum* leaves in control (C) and drought stress (DS) conditions. Relative expression values (*S. pennellii vs S. lycopersicum*) obtained by microarray (Log_2) and by RT-qPCR using the ΔΔCt method are compared in each experimental condition. RNA from leaflet tissue of plants grown in control condition was used as calibrator sample.

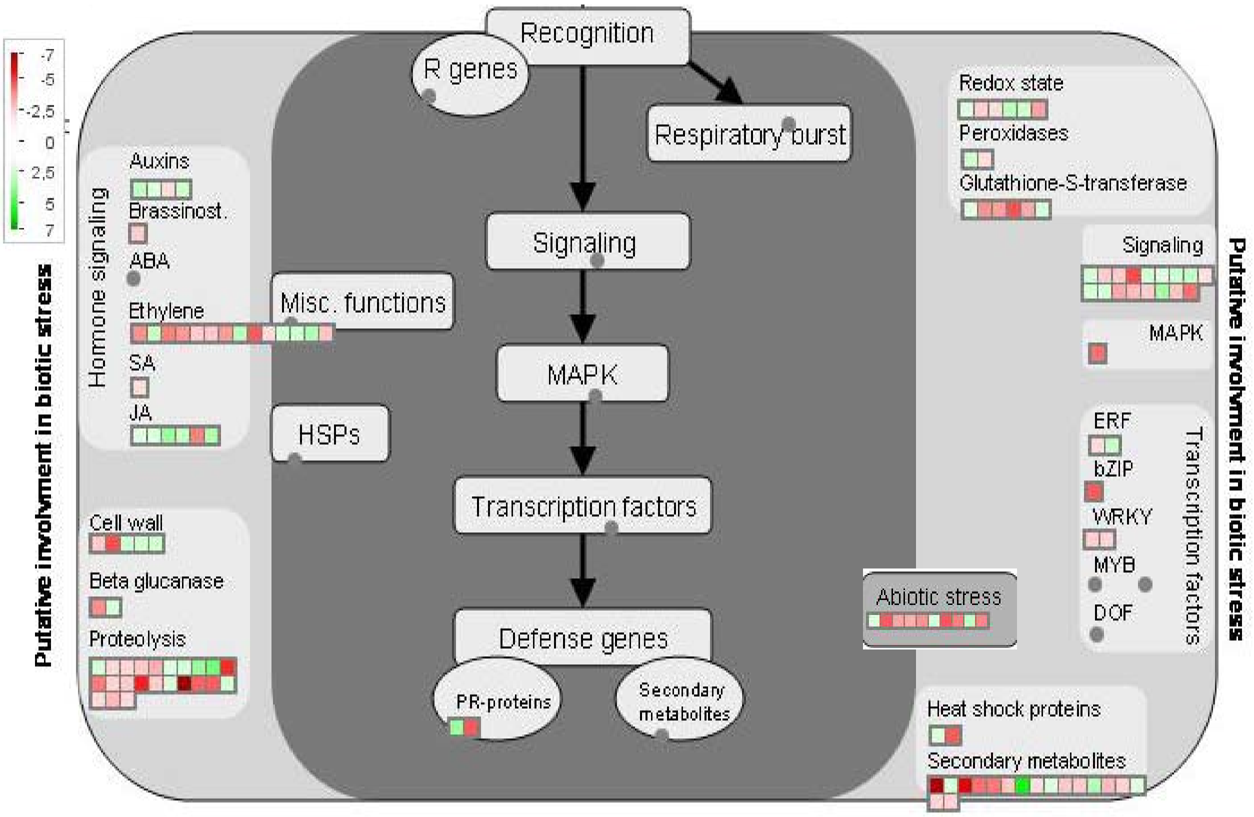

Supplementary Information Figure S2. Assignment of the 459 drought-responsive genes in the *S. pennellii vs S. lycopersicum* comparison to functional categories (bins) using the MapMan ontology Slyc_AFFY_SGN_BUILD2_070709 database (http://mapman.gabipd.org/web/guest/mapmanstore). Log_2 fold-change values between *S. pennellii* and *S. lycopersicum* are colour coded, red and green indicating decreased or increase gene expression. Note that the chosen general stress response map contains a majority of DEGs from the complete dataset (Supporting information Tables S1-S6). Differential gene expression was determined at FDR<0.05.

Supplementary Table S13. List of transcripts showing significant changes between *S. pennellii* and cultivated tomato in control and water stress, and between conditions in *S. pennellii*, reported in Results and Discussion sections. Apart from annotation/description the gene abbreviation is provided.

#	Probeset ID[1]	BinCode[2]	Gene ID[3]	Annotation/Description[4]	Abbreviation
**Acetyl-CoA production and isoprenoids biosynthesis**					
**1**	lesaffx.1956.1.s1_at	5.10	823955	aldehyde dehydrogenase 2B4	ALDH 2B4
**2**	les.2817.2.s1_at	8.2.11	842375	ATP-citrate lyase A-2	ACLA-2
**3**	les.2160.1.a1_at	11.1.20	825752	malonyl-CoA decarboxylase	MCD
**4**	les.4390.1.a1_at	16.1.2.2	826788	hydroxymethylglutaryl-CoA synthase	HMGS (MVA1)
**5**	Les.4457.1.s1_s_at	16.1.5	832461	Terpene synthase 21	TPS21
**Carotenoids biosynthesis**					
**6**	les.4438.1.a1_s_at	16.1.4.1	831587	phytoene synthase	PSY
**7**	les.2287.3.a1_at	29.6	830500	ORANGE-like protein	OR-L
***myo*-inositol biosynthesis & transport**					
**8**	les.2012.1.s1_at	3.4.3	830881	myo-inositol-1-phosphate synthase 3	MIPS3
**9**	les.3756.1.s1_a_at	34.2	818934	tonoplast myo-inositol exporter (H+/myo-inositol symporter)	INT1
**Photorespiration**					
**10**	les.2102.1.a1_at	1.2.2	820630	glycolate oxidase	GOX
**11**	les.1979.1.a1_at	7.1.3	838342	glyoxylate reductase 2	GLYR2
**Redox homeostasis**					
**12**	lesaffx.3918.1.s1_at	21.2.1	820121	cytosolic ascorbate peroxidase 2	APX2
**13**	lesaffx.51366.1.s1_at	11.8	835538	flavodoxin-like quinone reductase 1	FQR1
**14**	lesaffx.32653.1.s1_at	21.2.2	824631	gluthatione reductase 2	GR2
**15**	les.167.1.s1_at	21.6	817365	chloroplastic Cu/Zn superoxide dismutase 2	CSD2
**Lipid metabolism**					
**16**	les.745.1.a1_at	11.1.9	819337	long-chain acyl-coenzyme A synthase 1	LACS1
**17**	les.2095.1.a1_at	11.2.4	820387	ω-6-fatty acid desaturase 2	FAD2
**Pathogenesis-related and other stress-responsive proteins**					
**18**	les.3673.1.s1_at	26.4	824893	β 1,3-glucanase 2	BGL2
**19**	les.3583.1.a1_at	20.2.99	843890	salt tolerance homolog 2 transcription factor	STH2
**20**	lesaffx.823.1.s1_at	20.1	829514	putative pathogenesis-related protein	putative PR
**21**	les.1842.1.s1_at	20.1	840294	avirulence induced gene protein	AIG
**22**	les.5711.1.s1_at	29.5.3	830064	responsive to dehydration 19A transcription factor	RD19A
**Elongation factors**					
**23**	les.419.2.s1_at	29.2.4	836161	GTP-binding Elongation Factor Tu	EF-Tu
**24**	les.5230.1.s1_at	29.2.4	837491	elongation Factor 1B gamma	EF-1Bg

**Stomatal movement regulation**					
**25**	les.796.1.a1_at	8.3	821134	carbonic anhydrase 1	CA1
**Cell Wall metabolism**					
**26**	les.4530.1.s1_at	10.7	821955	xyloglucan:xyloglucosyl transferase xyloglucan endotransglucosylase/ hydrolase 9	XTH9
**27**	lesaffx.5130.1.s1_at	10.7	842879	expansin B2	EXPB2
**28**	lesaffx.32379.1.s1_at	26.2	838512	PARVUS Nucleotide-diphospho-sugar transferases superfamily protein (xylan biosynthesis, galacturonosyltransferase activity)	PARVUS/GLZ1
**Primary carbohydrate metabolism**					
**29**	les.1617.1.s1_s_at	2.1.1.3	840953	cytosolic fructose-1,6-bisphosphatase / FRUCTOSE INSENSITIVE 1	cFBP/FINS1
**30**	lesaffx.29037.1.s1_at	2.1.2.1	834883	ADP-glucose pyrophosphorylase 1	ADG1
**Nitrogen assimilation**					
**31**	les.2360.1.s1_at	12.1.2	816055	ferredoxin-nitrite reductase 1	NIR1
**32**	les.4317.1.s1_at	13.1.3.1	823888	glutamine-dependent asparagine synthase 1	ASN1
**33**	lesaffx.67643.1.s1_at	13.2.3.1	820860	asparaginase 2	Asparaginase 2
**34**	les.899.1.s1_at	30.1	835427	NADH-dependent glutamate synthase 1 / Glutamine-oxoglutarate aminotransferase	GLT1 (GOGAT)
**GDH- and GABA-Shunt**					
**35**	les.4851.1.s1_at	12.3.1	830635	glutamate dehydrogenase 2	GDH2
**36**	les.249.1.s1_at	13.1.1.1.1	831599	glutamate decarboxylase 1	GAD1
**37**	les.3289.1.s1_at	13.1.1.3	821784	gamma-aminobutyrate transaminase / POLLEN-PISTIL INCOMPATIBILITY 2	GABA-T (POP2)
**Amino acid transport**					
**38**	les.4075.1.s1_at	34.3	844074	amino acid permease 3	AAP3
**BCAAs metabolism**					
**39**	lesaffx.70406.1.s1_at	13.1.4.1.4	836707	branched-chain-amino-acid transaminase 5	BCAT5
**40**	lesaffx.69324.2.s1_at	13.2.6.3	836724	3-hydroxyisobutyryl-CoA hydrolase 1	CHY1
**ABA biosynthesis**					
**41**	les.3529.1.s1_at	34.99	817257	aldehyde oxidase 3	AAO3
**42**	les.3454.1.s1_at	18	838224	molybdenum cofactor sulfurase ABA3 (LOS5)	ABA3 (MCSU)
**JA biosynthesis**					
**43**	les.3632.1.s1_at	17.7.1.2	838314	lipoxygenase 3 (13-lipoxygenase)	LOX3
**44**	les.13.1.s1_at	17.7.1.3	834273	allene oxide synthase	AOS
**45**	lesaffx.38775.1.s1_at	17.7.1.5	844001	cis-(+)-12-oxo-phytodienoic acid reductase 2	OPR2
**46**	les.612.1.s1_at	17.8.1	838551	jasmonic acid carboxyl methyltransferase	JMT
**ET biosynthesis & signaling**					
**47**	les.5917.1.s1_at	17.5.1	816478	1-aminocyclopropane-1-carboxylate oxidase 1	ACO1

**48**	les.132.1.s1_at	17.5.1	839345	1-aminocyclopropane-1-carboxylate oxidase 4	ACO4
**49**	les.3818.1.s1_at	17.5.2	821902	ethylene-responsive element binding factor 1B	ERF1B
**50**	lesaffx.3059.1.s1_at	17.5.2	821900	ethylene-responsive element binding factor 095	ERF095

[1]ID of GeneChip® Tomato Genome Array probes
[2]Codes of functional categories as defined by MapMan bins (Thimm et al., 2004, Ref. 29) (http://mapman.gabipd.org)
[3]Gene ID from NCBI database (https://www.ncbi.nlm.nih.gov/gene)
[4]Description/functional annotation of each transcript derived from MapMan software (Thimm et al., 2004, Ref. 29) (http://mapman.gabipd.org)

Supplementary Table S14. Primers used in the expression analysis by RT-qPCR of selected differentially expressed genes for technical validation of the microarray data.

#	Probeset ID[1]	Gene ID[2]	Description/Annotation[3]	Abbreviation	Primer	5'-3'	Efficiency in *S. lycopersicum*	Efficiency in *S. pennellii*
1	les.419.2.s1_at	836161	GTP-binding elongation factor Tu family protein	EF-Tu	FWD	GCAGCCAAGGTTTAGCGAAG	137,80%	139,40%
					REV	GTCGACATGACCAATGACCAC		
2	les.5711.1.s1_at	830064	Responsive to dehydration 19A - cysteine proteinase	RD19A	FWD	CCTTCTGACGCCAATAAAGC	92,20%	89,90%
					REV	CGCATGAACCCTGATTCTTC		
3	les.5230.1.s1_at	837491	Elongation factor 1B gamma	EF-1Bg	FWD	TTCCAGATGGGTGTGTCAAA	102,30%	107,50%
					REV	CATAGCGTGCGATAGCATTG		
4	les.13.1.s1_at	834273	Allene oxide synthase	AOS	FWD	CAGGCTTCGGTGTCTGAGA	113,10%	108,40%
					REV	GACTTTTTGGGCTGGGAGTT		
5	lesaffx.3918.1.s1_at	820121	Ascorbate peroxidase 2	APX2	FWD	AGTGTTATCCGACGGTGAGC	127,20%	100,60%
					REV	TCTCAGCATAATCGGAGCAC		
6	les.2360.1.s1_at	816055	Nitrite reductase 1	NIR1	FWD	GTGGGCAAGCAATAATCGAG	87,90%	105,30%
					REV	CTGTCCAGTGCATCCTCACT		
7	les.249.1.s1_at	831599	Glutamate decarboxylase 1	GAD1	FWD	CGACGTCGATAAGAGATTCAGA	91,70%	100,50%
					REV	CTTCCTTCGGCATGGATTTT		
8	les.4317.1.s1_at	823888	Glutamine-dependent asparagine synthase 1	ASN1	FWD	GAGCTTTCTCGCAGGTTGAA	99,50%	106,70%
					REV	AGAAGCAGGGTCGATAATTGC		
9	les.4438.1.a1_s_at	831587	Phytoene synthase	PSY	FWD	CCCGTCGACTACGAAAAAGA	80,30%	79,30%
					REV	GTTTCTCATGCAAGGCGTAG		
10	les.2573.1.a1_at	818973	Fatty acid biosynthesis 2	FAB2	FWD	GGGGATGASTTTCTGGTGAGA	117,50%	98,80%
					REV	ACACACTAGGCAAGGGAAAAA		
11	les.4390.1.a1_at	826788	Hydroxymethylglutaryl-CoA synthase HMGS (MVA1)	MVA1	FWD	ATCGGAAATGGCTTCTCAACC	109,90%	113,60%
					REV	CTCCATCATGAGCCTCCAAT		

[1] ID of GeneChip® Tomato Genome Array probes
[2] Gene ID from NCBI database (https://www.ncbi.nlm.nih.gov/gene)
[3] Description/functional annotation derived from MapMan software (http://mapman.gabipd.org)
